# Limitations of variable number of tandem repeat typing identified through whole genome sequencing of *Mycobacterium avium* subsp. *paratuberculosis* on a national and herd level

**DOI:** 10.1186/s12864-015-1387-6

**Published:** 2015-03-08

**Authors:** Christina Ahlstrom, Herman W Barkema, Karen Stevenson, Ruth N Zadoks, Roman Biek, Rowland Kao, Hannah Trewby, Deb Haupstein, David F Kelton, Gilles Fecteau, Olivia Labrecque, Greg P Keefe, Shawn L B McKenna, Jeroen De Buck

**Affiliations:** University of Calgary, Calgary, Alberta Canada; Moredun Research Institute, Penicuik, Scotland; University of Glasgow, Glasgow, Scotland; SaskMilk, Regina, Saskatchewan Canada; University of Guelph, Guelph, Ontario Canada; Université de Montréal, Montréal, Québec Canada; Laboratoire d’épidémiosurveillance animale du Québec, Saint-Hyacinthe, Québec Canada; University of Prince Edward Island, Charlottetown, Prince Edward Island Canada

**Keywords:** *Mycobacterium avium* subspecies *paratuberculosis*, Variable number tandem repeat typing, Whole genome sequencing

## Abstract

**Background:**

*Mycobacterium avium* subsp. *paratuberculosis* (MAP), the causative bacterium of Johne’s disease in dairy cattle, is widespread in the Canadian dairy industry and has significant economic and animal welfare implications. An understanding of the population dynamics of MAP can be used to identify introduction events, improve control efforts and target transmission pathways, although this requires an adequate understanding of MAP diversity and distribution between herds and across the country. Whole genome sequencing (WGS) offers a detailed assessment of the SNP-level diversity and genetic relationship of isolates, whereas several molecular typing techniques used to investigate the molecular epidemiology of MAP, such as variable number of tandem repeat (VNTR) typing, target relatively unstable repetitive elements in the genome that may be too unpredictable to draw accurate conclusions. The objective of this study was to evaluate the diversity of bovine MAP isolates in Canadian dairy herds using WGS and then determine if VNTR typing can distinguish truly related and unrelated isolates.

**Results:**

Phylogenetic analysis based on 3,039 SNPs identified through WGS of 124 MAP isolates identified eight genetically distinct subtypes in dairy herds from seven Canadian provinces, with the dominant type including over 80% of MAP isolates. VNTR typing of 527 MAP isolates identified 12 types, including “bison type” isolates, from seven different herds. At a national level, MAP isolates differed from each other by 1–2 to 239–240 SNPs, regardless of whether they belonged to the same or different VNTR types. A herd-level analysis of MAP isolates demonstrated that VNTR typing may both over-estimate and under-estimate the relatedness of MAP isolates found within a single herd.

**Conclusions:**

The presence of multiple MAP subtypes in Canada suggests multiple introductions into the country including what has now become one dominant type, an important finding for Johne’s disease control. VNTR typing often failed to identify closely and distantly related isolates, limiting the applicability of using this typing scheme to study the molecular epidemiology of MAP at a national and herd-level.

## Background

*Mycobacterium avium* subsp. *paratuberculosis* (MAP) is the causative bacterium of Johne’s disease in ruminants and has significant impacts on the health of cattle and the economics of dairy production systems in particular [1]. This organism is primarily transmitted via the fecal-oral route and is generally introduced into herds through the purchase of one or more infected animals. Despite control efforts in the Canadian dairy industry, up to 76% of herds are infected with MAP [2]. Additionally, a potential association with Crohn’s disease in humans is increasing the pressure to control MAP on farms and in the environment [3,4].

Limited tools exist to assess within-species diversity of pathogens with low genetic variation [5], such as MAP. Despite this challenge, a number of molecular typing methods have been developed that target repeat regions in the genome and have been moderately successful in observing apparent diversity [6,7]. Variable number tandem repeat (VNTR) and mycobacterial interspersed repetitive unit (MIRU) typing are based on repetitive element differences and have been used in molecular epidemiological studies in a number of organisms and settings [8-12]. Most recently, Oakey et al. [13] used VNTR typing to investigate MAP incursions in a low-prevalence region of Australia and concluded that two separate VNTR types were circulating in this region. This genetic information was used to evaluate epidemiological links and inform policy decisions and control strategies.

To date, whole genome sequencing (WGS) of MAP has predominantly focused on *de novo* assembly of a limited number of relevant genomes [14-16]. While this has provided immense resources to understand the genomic structure and evolution of major strain types (types I, II, and III), WGS of many epidemiologically linked isolates can determine the true degree of diversity and quantify relatedness. Single nucleotide polymorphisms (SNPs) identified through WGS are evolutionarily stable and can be reliably used to identify true evolutionary relationships [17]. In a MAP-endemic environment, this level of detail is invaluable in understanding the molecular epidemiology and transmission dynamics.

Many reports have described the diversity of MAP isolates using VNTR typing with or without additional molecular targets [12,18-20]. The validity of using VNTR typing to help clarify transmission and the true diversity of MAP, however, has yet to be determined. Whilst the instability of repetitive elements contributes to their discriminatory ability, there is also the risk of convergent evolution, which may lead to incurred epidemiological inferences from VNTR data [21]. Therefore, the aim of this study was to assess the diversity of MAP isolates (type II) in Canadian dairy herds using WGS to assess the ability of VNTR typing to identify unrelated and related isolates at the national and herd level.

## Methods

### MAP isolates and DNA preparation

A collection of Canadian MAP isolates was derived from previously obtained samples through provincial Johne’s disease control programs and research projects. Positive culture broth from environmental manure samples was obtained from Johne’s disease control programs in Alberta and Saskatchewan [2], the Atlantic region (Prince Edward Island and New Brunswick) [22] and Québec (Table 1), while individual cow fecal samples were obtained from British Columbia and Québec. Six environmental manure samples were collected from participating Alberta and all Saskatchewan dairy herds. Additionally, individual cow fecal samples from cows with high milk ELISA titers in Ontario were obtained and cultured as described previously [23]. Samples from Québec were cultured using the BACTEC MGIT 960 ParaTB culture system (Becton, Dickinson and company, Franklin Lakes, NJ, USA), whereas the remaining samples were cultured using the TREK ESP Culture System reagents (TREK Diagnostics, Cleveland, OH, USA). Approximately 10 μl of culture broth was plated onto Middlebrook 7H11 agar supplemented with 2 mg/L mycobactin J to isolate individual MAP colonies. Plates were incubated at 37°C for 4–6 weeks. A single colony was subsequently substreaked onto 7H11 agar and incubated for 4–8 weeks prior to DNA extraction. DNA was extracted using a modified protocol of the Qiagen DNeasy blood and tissue kit (Qiagen, Mississauga, ON, Canada), as described previously [24].

**Table 1:** **Province of origin, number of herds, VNTR types and number of** ***Mycobacterium avium*** **subsp.** ***paratuberculosis*** **isolates analyzed**

**Province/Region**	**# Herds**	**# VNTR typed**	**VNTR types (INMV #)**	**# Whole genome sequenced**
Alberta	98	300	1, 13, 17, 2, 3, 6, 68, 77, NEW2	58
Atlantic regions	10	62	17, 2, 77	11
British Columbia	8	14	2, 3	5
Ontario	17	52	17, 2, 3, 6	16
Quebec	24	45	1, 2, 3, 68	14
Saskatchewan	25	54	1, 2, 3, 6, 7, 117, NEW	20
Total	182	527		124

### VNTR typing

VNTR typing targeting eight loci was performed on 527 DNA samples extracted from MAP isolates using a previously established protocol [7] and VNTR types (INRA Nouzilly MIRU-VNTR [INMV] profile number) were assigned according to the MAC-INMV database [25].

### Whole genome sequencing

MAP isolates were selected for WGS to 1) represent the approximate proportion of isolates per VNTR type in Canada (while minimizing duplicate types from the same herd), 2) include all Canadian provinces currently represented in the MAP collection, and 3) represent three different scenarios of VNTR type diversity at the herd-level in Alberta. The three Alberta herd-level isolate sample sets included a herd with (A) four isolates of the same VNTR type, (B) three isolates with three different VNTR types, and (C) four isolates with two different VNTR types. As these isolates were cultured from environmental manure samples, true herd-level diversity was not assessed.

MAP DNA was prepared for sequencing using the NexteraXT sample preparation kit (Illumina, San Diego, CA, USA). Samples were multiplexed to achieve paired end reads with an average coverage of 50X and sequenced using V2 (250 bp reads) or V3 (300 bp reads) chemistry using the Illumina MiSeq sequencing platform (Illumina, San Diego, CA, USA).

### Data analysis

Raw reads were trimmed using ConDeTri [26] and mapped to a revised version of the K10 reference genome (NCBI Sequence Read Archive study SRR060191) [27] using BWA [28]. Variant sites were identified using SAMtools [29] and were subsequently filtered based on depth of coverage (at least 2 high quality SNPs on both the forward and reverse strand), mapping quality (>40), and heterozygosity (<5%). SAM files were visualized in Tablet [30] to investigate specific polymorphisms of interest, such as the 2 base-pair deletion in IS1311 “bison type” isolates [31]. An alignment of concatenated SNPs was used to create a maximum likelihood phylogenetic tree using PhyML [32], using the nucleotide substitution model, as determined by jModelTest [33]. Clade support was evaluated based on the analysis of 100 bootstrap pseudo-replicates. To determine how specific VNTR loci contribute to the phylogenetic congruence, a circularized phylogenetic tree was annotated according to the six loci that were polymorphic in the WGS dataset (292, X3, 25, 47, 7, and 10) [34].

Additionally, the absolute minimum, maximum, and average pairwise genetic distance of each isolate to every other isolate within the same VNTR type and between VNTR types was calculated for the three most common types by averaging across the PhyML generated distance matrix. To identify how frequently closely related isolates differ in VNTR type, the number of pairwise distances smaller than a range of cut-off values (<5, <10, <15, <20, <25, <30, <35, <40, <45, <50, <75, <100, <200) was calculated within and between types.

## Results

### VNTR typing

MAP isolates evaluated in this study came from a total of 201 Canadian dairy herds from British Columbia, Alberta, Saskatchewan, Ontario, Québec, and the Atlantic region. VNTR typing was performed on 527 MAP isolates and resulted in 12 different types (Table 2). A total of 76, 8, and 7% of MAP isolates belonged to INMV type 2, 3, and 1 respectively, with 9 types representing the remaining 9% of isolates. Two loci (3 and 32) were not found to be polymorphic, while loci 292, X3, 25, 47, 7, and 10 contained 3, 3, 3, 2, 2, and 2 alleles, respectively. MAP isolates belonging to INMV68, which was previously identified as “bison type” isolates in Québec [18], were identified in seven herds (two from Québec and five from Alberta) (Table 1).

**Table 2:** **The number of MAP isolates, herds, and whole genomes sequenced (WGS) within each VNTR type**

**VNTR type**	**Locus**								**# isolates**	**# herds**	**# WGS**
	**292**	**X3**	**25**	**47**	**3**	**7**	**10**	**32**			
INMV 2	3	2	3	3	2	2	2	8	398	139	83
INMV 3	3	2	3	3	2	2	1	8	41	25	16
INMV 1	4	2	3	3	2	2	2	8	36	16	13
INMV 17	3	1	3	3	2	2	2	8	18	3	3
INMV 68	2	2	5	3	2	2	2	8	17	6	1
INMV 13	2	2	3	3	2	2	2	8	6	2	1
INMV 6	3	2	3	3	2	1	2	8	4	4	2
INMV 77	3	2	2	3	2	2	2	8	3	2	2
INMV 7	3	2	3	3	2	1	1	8	1	1	1
INMV117	3	2	3	2	2	2	2	8	1	1	1
NEW1	3	3	3	3	2	2	1	8	1	1	1
NEW2	4	2	3	2	2	2	2	8	1	1	0

### Whole genome sequencing

**Figure 1:**
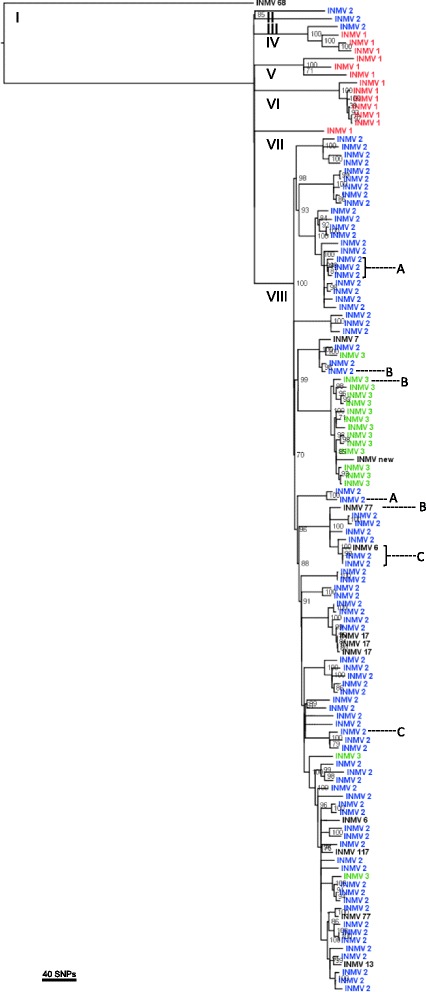
**Maximum likelihood phylogenetic tree created from 3,039 concatenated variant sites using PhyML and the TPM1uf nucleotide substitution model, rooted to the “bison type” isolate (INMV 68).** The tips are labeled as the VNTR type and the three most prevalent types are color-coded (INMV 2 = blue, INMV 3 = green, INMV 1 = red). Dotted lines indicate samples belonging to Herds A, B, and C, the eight divergent subtypes are labeled I-VIII, and bootstrap values of node support ≥ 70 are displayed.

124 isolates were successfully sequenced, representing 11 different VNTR types, with an average coverage of 33X. 3,039 high quality variant sites were identified and used to create a maximum likelihood phylogenetic tree (Figure 1). Whole genome sequencing revealed more than 650 SNPs unique to the “bison type” isolate, and visualization of the reads in Tablet confirmed a ‘TG’ deletion unique to “bison type” strains in the IS13111 sequences. Eight divergent subtypes were identified that contained at least 45 unique SNPs relative to the most recent common ancestor of all isolates, excluding the “bison type” outgroup. The dominant subtype (subtype VIII) included 86% of isolates analyzed.

### Analysis of VNTR typing and WGS

In Figure 1, tips of the phylogenetic tree were labeled according to VNTR type and the three most common types were color-coded. INMV 2 isolates in this dataset originated from all six provinces, whereas INMV 3 and INMV 1 isolates were identified from five and three provinces, respectively. Of the 11 VNTR types identified, nine were located within the dominant subtype (VIII).

**Figure 2:**
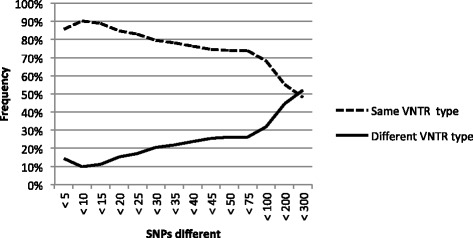
**The frequency in which any two MAP isolates share the same VNTR type (solid line) or different VNTR types (dotted line) at a range of pairwise SNP differences (<5 to <300 SNPs) (i.e. two isolates that have fewer than 300 pairwise SNP differences belong to different VNTR types 52% of the time).**

The frequency with which isolates differing by a range of SNPs belong to different VNTR types is depicted in Figure 2. In this dataset, MAP isolates differing by <10 SNPs belonged to different VNTR types 10% of the time. This value increased as the number of SNP differences increased.

**Figure 3:**
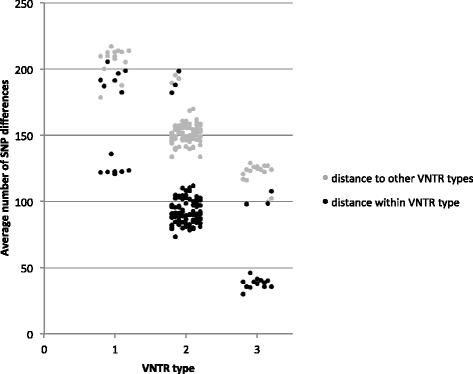
**Scatter plot of the average pairwise SNP distance, as determined by the maximum likelihood distance matrix, between each MAP isolate within INMV 1, INMV 2, and INMV 3 (black) and between those VNTR types and isolates of all other VNTR types identified in this study (grey) based on 8 VNTR loci.**

The average number of pairwise SNP differences between isolates within the three most common VNTR types and outside of each type is presented in Figure 3. The majority of isolates within a type had lower average SNP differences when compared to the differences between VNTR types (Figure 3). However, in each of the three major VNTR types, several isolates with within-type differences clustered with the between-type differences, with average SNP differences that were considerably higher than would be expected for isolates of the same VNTR type. INMV 3 isolates were on average more closely related, with less than 50 SNP differences in the largest cluster, compared to INMV 2 isolates, which were approximately 100 SNPs different. INMV 1, on the other hand, had on average over 150 SNP differences between isolates within that type, with one isolate exceeding a difference of 200 SNPs. The absolute maximum number of SNP differences between any two isolates within each type was 240, 215, and 116 for INMV 1, 2, and 3 respectively, while the minimum number of SNP differences was one for INMV 1 and INMV 2 and four for INMV 3 (Table 3). The absolute maximum and minimum number of SNP differences between two different VNTR types for INMV 1, 2, and 3 was also calculated. In the case of INMV 1, the maximum SNP difference within the type was larger than between types (Table 3). The dominant subtype included five different VNTR types that differed from INMV 2 by 15 SNPs or less, including INMV 17 that differed from an INMV 2 isolate by only two SNPs (Figure 1).

**Table 3:** **Absolute minimum and maximum pairwise SNP difference within and between the three major VNTR types**

	**INMV 1**		**INMV 2**		**INMV 3**	
	**Within**	**Between**	**Within**	**Between**	**Within**	**Between**
Absolute minimum	1	70	1	2	4	15
Absolute maximum	240	239	215	236	116	230

Three herds with multiple MAP isolates per herd were evaluated using VNTR typing and WGS. Four isolates of the same VNTR type were analyzed in Herd A, three isolates of different VNTR types were analyzed in Herd B, and four isolates representing two VNTR types were analyzed in Herd C. Herd B VNTR typing results correctly differentiated distantly related isolates; however, isolates from Herd A and Herd C had inconsistent typing results (Figure 1) with either unrelated isolates belonging to the same VNTR type (Herds A and C) or different VNTR types being closely related (Herd C).

**Figure 4:**
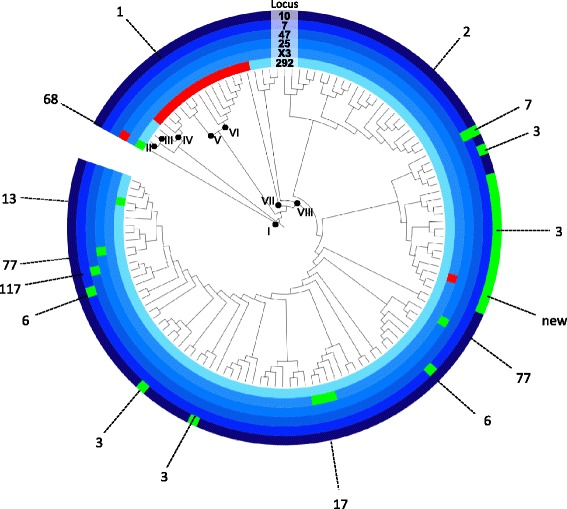
**Circularized maximum likelihood phylogenetic tree with each polymorphic VNTR locus concentrically displayed (inner to outer: 292, X3, 25, 47, 7, and 10).** Branch lengths are not shown to scale. Blue represents the most common repeat number within each locus, red indicates a larger repeat number, and green represents a smaller repeat number. A dotted line indicates the VNTR type that represents each locus combination and the subtype (I-VIII) of each isolate/clade is indicated by a black dot located at the ancestral node of the isolate(s) within that subtype.

Additionally, there was evidence of convergent evolution in four of six VNTR types in which more than one isolate per type was sequenced (INMV 2, 3, 6, and 77) (Figure 1). Locus-specific changes in repeat length are presented in Figure 4. Locus 10 had the most instances of convergent evolution (five times), whereas loci 47 and X3 appeared to be relatively stable.

## Discussion

In this study, the genetic relationship of 124 Canadian bovine MAP isolates was assessed using WGS and VNTR typing. Main findings were that 1) at least eight genetically distinct MAP subtypes exist in Canada, with over 80% of isolates belonging to a single dominant subtype, and 2) VNTR typing may both overestimate and underestimate relatedness. Additionally, evidence of convergent evolution was observed multiple times and individual VNTR loci contributed differently to the levels of VNTR-level homoplasy observed in the SNP-based phylogeny.

The phylogenetic analysis of MAP isolates based on over 3,000 SNPs identified through WGS revealed eight divergent subtypes, one of which contains over 80% of isolates. The presence of a dominant subtype in Canada could be attributed to a variety of factors, such as increased virulence, increased culturability, or a founder effect (e.g. the first MAP introduction into Canada). The branch lengths separating the major subtypes exceed 100 SNPs, suggesting there were likely at least eight separate introduction events into Canada, as the accumulation of this number of SNPs is unlikely within the timeframe of Holstein-Friesian dairy farming in Canada (1881 to the present) [35].

In the absence of observational data, a suboptimal typing technique will falsely classify related isolates as unrelated, masking the true relationship, or overestimate relatedness, incorrectly linking unrelated isolates. Within the three most common VNTR types, some isolates were highly unrelated based on the number of SNP differences, with as many as 240 differences within a type (INMV 1). Given the slow mutation rate of MAP, estimated to be slower than the 0.3 SNPs/genome/year in *Mycobacterium tuberculosis* ([36]; Bryant et el., unpublished), these isolates are highly unrelated. Falsely classifying isolates as related is characteristic of a typing technique with a low discriminatory ability, which can be resolved by including additional discriminatory genetic markers.

In contrast, this dataset provides examples where evidence of transmission based on VNTR typing would be lost due to a change in repeat number at a single locus (i.e. INMV 2 and 17; INMV 2 and INMV 6). This has similarly been demonstrated in the evaluation of VNTR typing of *M. bovis*, where single locus variants were present in the same herd, suggesting the presence of clonal variants [37]. While it has been demonstrated that genetic data based on repetitive elements are not appropriate for deep phylogenetic inference [5], the observation that a VNTR type can be identical by convergent evolution indicates that classifying isolates based on VNTR type may lead to erroneous epidemiological conclusions regarding transmission events occurring within a timeframe of a few years. Given that the most severe cases of convergent evolution occur in the dominant subtype (VIII), the use of a typing scheme that first defines major lineages based on SNPs followed by VNTR and/or additional typing methods, as has been proposed for other organisms [5,9], will not substantially clarify transmission dynamics at this limited spatial scale.

The analysis of three herd-level datasets illustrates the value of using WGS data for the study of epidemiologically linked isolates and that VNTR typing data may lead to an incorrect assessment of diversity and relatedness of strains. Herd A harbored at least two different strains that appear to be identical based on VNTR typing. Herd C also had at least two different strains circulating; however, despite the presence of two different VNTR types, presence of INMV 6 is likely due to within-herd evolution of the closely related INMV 2 isolates, whereas the more divergent INMV 2 isolate was probably the result of a separate introduction event.

Locus-specific differences in repeat length were also evaluated. Convergence was observed in four of six loci and overall a higher frequency of loss, rather than gain, of repeat numbers relative to the most common allele at each locus was found, which has been observed previously in *M. tuberculosis* [38,39]. Prediction models for VNTR locus evolution in other organisms have been developed [21,40,41], but they have not been based on comparison with WGS data. It has been reported previously [9] that different genetic markers should be selected for different spatial and temporal scales, so that the molecular clock speed of the marker is matched to the scale of the investigation. However, results from the current study suggest that the current VNTR typing scheme includes loci that are too unstable, and therefore unreliable, to be used for molecular epidemiological analysis of MAP at both broad and limited spatial scales.

## Conclusions

The usefulness of molecular typing strongly depends on its ability to differentiate epidemiologically meaningful differences between isolates. Molecular epidemiological studies of MAP have not yet provided sufficient results to truly impact our understanding of the transmission dynamics and genotype-specific virulence characteristics. Correctly classifying strains as “same” or “different” is important for transmission studies and in identifying phenotypic associations. Based on results from this study, caution should be used when using VNTR typing as a tool to assess the diversity and relatedness of MAP isolates at both a national and herd-level. Whole genome sequencing, on the other hand, provides unparalleled detail regarding the genetic profiles of MAP. The dominance of a single clade of MAP in Canada shows how important it is to have such detailed information when attempting to use molecular data to study MAP transmission dynamics.
